# The Impact of Parkinson’s Disease on Postural Control in Older People and How Sex can Mediate These Results: A Systematic Review

**DOI:** 10.3390/geriatrics6040105

**Published:** 2021-10-29

**Authors:** Mathieu Dallaire, Guillaume Gagnon, Émilie Fortin, Josée Nepton, Anne-France Severn, Sharlène Côté, Suhaila Mahmoud Smaili, Hayslenne Andressa Gonçalves de Oliveira Araújo, Márcio Rogério de Oliveira, Suzy Ngomo, Julie Bouchard, Rubens A. da Silva

**Affiliations:** 1 Masters of Biomedical Science Program at l’Université du Québec à Chicoutimi (UQAC), Saguenay, QC G7H 2B1, Canada; mathieu.dallaire2@uqac.ca (M.D.); Suzy_Ngomo@uqac.ca (S.N.); 2 Laboratoire de Recherche BioNR, Centre Intersectoriel en Santé Durable, Département des Sciences de la Santé, Université du Québec à Chicoutimi (UQAC), Saguenay, QC G7H 2B1, Canada; Julie1_Bouchard@uqac.ca; 3 Physical Therapy McGill Program in Extension at the Université du Québec à Chicoutimi (UQAC), Saguenay, QC G7H 2B1, Canada; guillaume.gagnon2@uqac.ca (G.G.); emilie.fortin7@uqac.ca (É.F.); 4 Centre Intégré de Santé et Services Sociaux du Saguenay—Lac-Saint-Jean (CIUSSS SLSJ), Specialized Geriatrics services–La Baie Hospital, Saguenay, QC G7H 7K9, Canada; josee.nepton.ccc@ssss.gouv.qc.ca (J.N.); anne-france.severn.labaie@ssss.gouv.qc.ca (A.-F.S.); shar.cote@gmail.com (S.C.); 5 Doctoral Program in Rehabilitation Sciences, UEL/UNOPAR, Londrina 86041-120, Brazil; suhailaneuro@gmail.com (S.M.S.S.); H.andressa.GOA@hotmail.com (H.A.G.d.O.A.); 6 Doctoral and Masters Program in Physical Exercise on Health Promotion, Universidade Pitagoras UNOPAR, Londrina 86041-120, Brazil; marxroge@hotmail.com

**Keywords:** motor control, postural control, postural instability, balance, aging, Parkinson’s disease, rehabilitation, sex

## Abstract

Introduction: Parkinson’s disease is most prevalent among elderly people, 65 years and over, and leads to an alteration in motor control associated with postural instability. Current evidence shows that postural control decreases with the aging process. In addition, postural control is more altered in healthy aged men than in women. Until today, few studies have evaluated the combined impact of Parkinson’s disease and sex on postural control. This review has allowed to evaluate the impact of Parkinson’s disease and sex on postural control measurements in elderly people. Methodology: Studies have been selected from two main databases: PubMed and EBSCO using the keywords “Parkinson”, “postural control OR balance” and “sex”. Articles related to the evaluation of postural control, including men and women with Parkinson’s aged over 65 years old, regardless of stage, were included (*n* = 179). Articles were excluded if not written in French or English or not presenting original content. Results: Ten (10) studies out of 179 that fulfilled inclusion and exclusion criteria were reported in the final analysis, which cumulates a total of 944 individuals with Parkinson’s (410 women). In general, results show greater postural instability among people with Parkinson’s compared to healthy subjects, and this according to different objective measurements using stabilographic parameters from force platforms. Only two studies out of ten evaluated postural control while briefly considering distinctions between sex, but without showing a significant difference between men and women with Parkinson’s. Parkinson’s severity, length of time of Parkinson’s disease and cognitive state of the person are the three variables with a negative impact on postural control. Conclusion: Older people with Parkinson’s disease have greater postural instability. Sex does not seem to influence the postural control of elderly people with Parkinson’s, although more studies are necessary.

## 1. Introduction

The proportion of people aged 65-years-old and over is increasing, worldwide. When following this progression, these people should represent 23.1% in North America by 2031, like the Canadian population [1]. With the natural process of aging, chronic degenerative diseases are more prevalent and further impact the biological changes associated with the proper aging process, such as muscular weakness, mobility impairment and poor balance or postural control [2,3,4]. These changes may predict frailty in the older individual as well as an increase in the risk of falls in this population [5,6,7]. Furthermore, poor postural control is one of the most important risk factors of falls in older adults [8]. Falls can be responsible for 85% of hospitalizations with injury in the elderly [9]. They are associated with multiple injuries and disabilities [10,11,12] as well as physical injuries, social isolation, psychological problems (anxiety, fear and depression) and increased mortality rates [12,13,14,15].

Among chronic and degenerative diseases in older adults, Parkinson’s disease is one of the most prevalent in individuals aged 65 years and over [16,17]. Moreover, the incidence of this disease would be two times higher in older men than in older women [18]. Parkinson’s disease involves a malfunction of the dopaminergic mechanism in the central nervous system [7,19]. As we know, dopaminergic neurotransmitters are essential in the production of coordinated movement [20,21]. Therefore, due to deficiencies in the dopaminergic system, this disease is characterized by motor symptoms such as stiffness, bradykinesia, tremors, coordination deficits and poor balance [20,21,22]. The progression of Parkinson’s disease can be considered according to the severity of symptoms [12,23], such as more important balance, speech, autonomic, cognitive and psychological disorders in more advanced stages [24]. Pharmacologic, surgical, psychological treatments and rehabilitation exist to alleviate the symptoms and limitations caused by the disease, associated or not with the effect of aging, but none aim to recovery [25].

Some evidence reported an impairment of balance among patients due to Parkinson’s disease [12,26,27], and this phenomenon, associated or not to the aging process, often, affects motor function, vision, vestibular system and proprioception of postural control [21,28,29]. In addition, a study reports worse postural control in the older adults with Parkinson’s versus the young Parkinson’s group [30]. However, few studies have assessed the impact of sex on postural control and health condition in older Parkinson individuals [31,32]. The results from these two studies were not completely related to force platform measurements only. In fact, Tassorelli et al. [31], which evaluated the functional balance of 42 individuals suffering from idiopathic Parkinson’s disease (mild-to-moderate severity), reported a greater degree of osteoporosis in their participants males than females (Z-score: M −3.8 ± 1.6, respectively) and that women fell more than men (fallers: 20 F/7 M; non fallers: 4 F/11 M), but not directly comparing the balance themselves. The second study [32] assessed the postural sway during quiet stance with eyes opened and eyes closed under force platform in 28 individuals with Parkinson’s disease and in 32 age- and sex-matched control subjects. The amount of postural sway in the patients was greater than in the control subjects, the higher level being most marked in patients with severe or long-duration of disease. The sex-related to differences on balance were again not reported for these authors. When looking for other types of disease (*ex: respiratory), apparently older women show better postural control under center of pressure measurements with a force platform than men in the same age group [33]. Wolfson et al. [34] (*n* = 234) demonstrated, however, that elderly healthy women show balance impairments when deprived of somatosensory and visual inputs and tend towards falling compared to elderly men. 

To summarize, the results are controversial, as only from one population and task-dependent when the sex is of concern [35,36]. It would be interesting to know the actual profile of the literature review with regard to postural control based in force platform measurement in older people with Parkinson’s disease and if sex can really mediate the results. Thus, the main purpose of this study was to highlight the state-of-the-art literature regarding the results of the impact of Parkinson’s disease on postural control in older people and to determine if the person’s sex as well as other factors can mediate these results of balance. 

## 2. Methodology

This systematic review (not related to a registered protocol) was conducted in alignment with checklist from Preferred Reporting Items for Systematic Reviews and Meta-Analyses (PRISMA).

### 2.1. Search Strategy and Studies Selection

**Figure 1:**
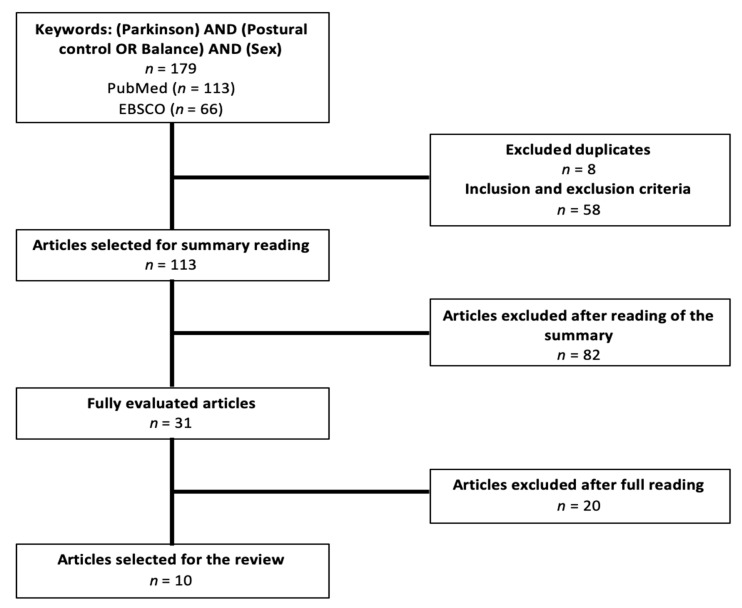
Articles selection process.

The search was conducted in the form of a search until the years 2020. Two main databases were consulted to conduct this research: PubMed and EBSCO. Various combinations of keywords were used to minimize the risk of selection bias. In general, the following strategy was used: (“Postural Balance”[Mesh] OR “Force plat*” OR postural control OR balance) AND (“Aged”[Mesh] OR elder* OR age* OR Aging) AND (“Parkinson disease”[Mesh] OR “Parkinson” OR Parkinson older) AND (“Sex”[Mesh] OR Gender* OR Men* OR Women). In each database, filters for language (French and English), species (human) and without final publication date were applied. A total of 179 studies were found and analyzed for the first step of this research (see Figure 1).

Two reviewers carried out the initial search strategy in the databases, extracting the titles and abstracts. Subsequently, the selection of studies, evaluation, and data extraction, was conducted independently by two evaluators (two physiotherapists), based on the reading of titles and abstracts. Potentially eligible articles were read in full. A manual search was performed in the reference lists of all eligible articles, to find new references. Disagreement between physiotherapist evaluations was resolved through discussion or by consulting a third review author (RDS). The same form for data extraction was used by the authors.

The selection criteria included randomized and non-randomized studies: (i) includes a Parkinson aging population (≥65 years old), [32] includes postural control measurement from force platform, and (iii) men and women in the assessment. The articles were excluded if (i) written in a language other than French or English, [32] they did not present original content (i.e., literature reviews).

The duplicates (*n* = 8) and articles not respecting inclusion/exclusion criteria (*n* = 58) were removed to obtain a total of 113 articles corresponding to the research subject. Articles not related with this research subject or containing exclusion criteria were also removed as illustrated in the Table 1. The evaluators also elaborated a list of abbreviation with variables for help in the selection (Table 2). Then, a reading of the entire articles was done (*n* = 31) and exclusion criteria were applied. An evaluation of study quality was done, namely using the “Quality Assessment Tool for Studies with Diverse Designs” [37] and was finally reported in Table 3. Only 10 articles were ultimately selected for the literature review after final analysis as illustrated in the Figure 1.

### 2.2. Data Extraction

First, a table reported the characteristics of Parkinson’s participants in the review such as age, sex, Hoehn and Yahr stage of disease and the number of participants for studies analyzed is shown in Table 4. Afterwards, the results for each study were compiled in table form, including the different groups, measurement tools used, goal of the study and main results of the literature (Table 5). 

## 3. Results

Ten (10) articles were analyzed in this literature review, cumulating 944 participants (534 men and 410 women) with a diagnosis of Parkinson’s disease (*n* = 665) and 226 participants in the control group. The quality of this study was around 22.1/42 ± 3.2 according to QATSDD, representing a moderate quality. All these studies were based in cross-sectional design so that without to establish the cause-effect on issue.

It is important to note that the two studies by Galna [38,39] included the same population so we included these results only once. The participants’ characteristics can be found in Table 4, including participants mean age which is 69.1 ± 3.7 years old and the duration of the Parkinson’s diagnosis which varies from 1 to 15 years. From the 10 studies, eight studies observed a Mini Mental State Examination (MMSE) score over 24/30, which included older people with Parkinson’s with cognitive impairment ranging from none to light mental state. A total of 534 men and 410 women took part in these studies. We observed that few studies briefly compared men and women (only two) and, across these, no statistically significant results were present regarding the impact of sex on Parkinson’s disease [31,32]. However, women in the study by Tassorelli et al. [31] demonstrated a lower level of functional independence (FIM = 98.9 ± 15.3 for men and 91.9 ± 16.6 for women), a lower Berg Balance Scale score (BBS = 51.9 ± 11.7 for men and 43.2 ± 12.8 for women) compared to men and a greater fear of falling than men (FES = 10.1 ± 6.7 for men and 20.9 ± 9.9 for women). Apparently, these results were not generalized for force platform measurements.

In addition, many measurement tools have been used among the selected studies to analyze balance and postural control of the participants, such as the GAITRite system, the Vicon, the Vitaport Activity Monitor, force platforms and other standardized tests or measurements (Berg Balance Scale, timed up and go, Unified Parkinson’s Disease Rating Scale, and Hoehn and Yahr Scale). Some studies have also used the inclinometer as a measurement tool of postural instability (Table 4 and Table 5). From these studies, Parkinson’s patients reported poor postural control when compared to those in control groups. It is also observed that severity [32,38,40] and cognitive state [41,42,43] for these patients were other factors that negatively affect postural control, but these studies do not report any differences between the sexes. 

Galna et al.’s study (2013) [39] has shown that patients with Parkinson’s disease and with more severe motor impairments have higher postural instability during walking and striding an obstacle compared to the control group. Marchese et al. [41] that analyzed the variation of the pressure center on a force platform under different conditions, reported significant difference between-groups (Parkinson’s group vs. control group) in the variation of the center of pressure during the execution of a double task (associating cognitive and motor skills). Rochester et al. [44] 48also noticed some changes during double tasks (cognitive and motor) such as a decreasing walking speed in Parkinson’s group, supporting the impact of attentional capacity on dynamic balance during walking. 

Viitasalo et al.’s study [32], that focused on postural balance with an inclinometer, noted a poor control in patients with a more severe stage of the disease as compared to the control group, without contrasting any differences in sex factor. Morris et al.’s study [45] demonstrated that patients with Parkinson’s disease have more difficulty keeping their balance during simple tasks, double tasks, verbal cognitive tasks, and when the base of support is reduced. 

Finally, two studies including Parkinson’s over 10 evaluated the two sexes (males and females) from balance measures (graph length of COP and/or COP sway by force platform or inclinometer). Both studies [31,32] reported no direct comparison between-sexes, while only Viitasalo et al. [32] showed that men and women are comparable on postural control during force platform measurement. 

## 4. Discussion

The aim of this study was to evaluate the impact of Parkinson’s disease on postural control in older adults and verify if sex can mediate the results. An update of the literature was necessary because the last review on this topic was in 2018, even searching up to 2020.

Although balance impairment in Parkinson’s is well known, only 10 articles were selected from this topic for analysis in older people and in sex differences related to balance. The impact of the disease on balance will be discussed first, followed by the impact of sex. In general, Parkinson’s disease alters the postural control of aged people and results are generalized for both sexes (*n* = 2 studies that compared the effect of sex on postural measures).

### 4.1. Impact of Parkinson’s Disease

During the process of aging, many factors are associated with a decrease in postural balance, such as the decrease of muscle mass and strength and the alteration of visual, vestibular, and proprioceptive systems [2,4,21,28,29]. It seems that age contributes to a transition between automatic postural control and an attentional, slowing down reaction rate to imbalance [4,41,45]. Parkinson’s disease exacerbates these effects [20,21,22]. Indeed, the literature demonstrates that the decrease in the secretion of dopamine in the substantia nigra in the brainstem is responsible for the main motor symptoms associated with Parkinson’s disease [20,21,22]. The decrease in postural control would be due to several factors, in particular by the increase in rigidity caused by the decrease in neurotransmitter production, which makes the trunk less flexible to disturbances therefore rendering the person with the disease more susceptible to be destabilized [20,21,22,29,46]. A decrease in postural reflexes would also be associated with this phenomenon [21,29,46]. However, although taking Levodopa reduces the severity of symptoms, it is not sufficient to eliminate them, suggesting that other structures may be responsible for the decrease in postural control in Parkinson’s patients [21,47,48].

Indeed, cholinergic cortical denervation would be an important marker of the slowing of walking in Parkinson’s disease [49]. Additionally, another post-mortem study on monkeys and patients with the disease demonstrated the importance of the role that cholinergic neurons of the pedunculopontine nucleus play walking and postural control deficits [48]. Damage to these structures would be, like the dopamine deficit, responsible for the appearance of rigidity, a reduction in stride length and a recurved posture, this affecting the patients’ ability to regain balance, in the event of a disturbance of the center of gravity [21,29,48]. Abnormalities would be more marked in people with an increased severity of the disease, explaining why anti-parkinsonian medication is not sufficient in these patients [21,47].

From the article selection, some studies evaluated the postural control by objective measurement using different tools, such as a force platform and GaitRite system to discriminate the differences between the Parkinson’s group versus the healthy-control group. A study of Tassoreli et al. evaluated stability with a stabilometric platform. Results showed significantly higher values (*p* < 0.001) for the Parkinson’s group compared to the control group for the graph length (1303.9 ± 656 mm vs. 987.3 ± 356 mm), amplitude of antero-posterior (29.7 ± 21.6 mm vs. 16.2 ± 6.4) and transverse oscillations (33.4 ± 17.1 mm vs. 15.2 ± 9.3 mm). In general, these studies showed a difference regarding postural oscillations in people with Parkinson’s disease compared to the control group when evaluated with a vertical inclinometer or placed at the waist [32,43], with a force platform [39] or with a motion analysis system in 3D VICON [32]. Indeed, balance is more affected in people with Parkinson’s than in healthy people during a static standing posture and during double tasks (opened and closed eyes, countdown, motor task: Thumb to fingers) on a force platform [31,41] or with other measurement methods [39,45]. These results are partly explained by the fact that people with Parkinson’s use conscious attentional strategies to compensate the impairment of basal ganglions, saturating their attention and making cortical control less efficient, highlighting balance impairments, especially during double tasks [41]. 

It is the same with internal perturbations, such as climbing over an obstacle, or external ones, which suggests that people with Parkinson’s disease have more difficulty managing disturbances on their center of mass and, therefore, more difficulty correcting their center of pressure to maintain balance [38,39]. Experimental balance conditions such as tandem or semi-tandem and one-legged stance tasks could discriminate a better impact of the disease on postural control than simple balance conditions, such as bipodal. In addition, unilateral impairment from the disease can affect the capacity to maintain such leg positions during balance performance [45]. Only the study of Tassorelli et al. [31] observed non-significant differences on oscillation speed, but range and length of range were higher in Parkinson’s disease people. These results seem to indicate that affected people oscillate at the same speed as the other group, but they take more time to react, which increases their oscillation distance. 

The duration of the disease would have an impact on performance during different balance tasks [32,45]. Indeed, the duration of the disease (>5 years) would have a strong correlation between the increase of range and velocity of postural oscillations resulting in increased postural instability. However, it seems that severity would have a higher impact than duration of the disease on postural balance. Hiorth et al. [40] evaluated people with new and old diagnoses of Parkinson’s disease and a history of falls. This study concluded that fallers had a more severe manifestation of Parkinson’s disease. This conclusion suggests that postural instability would be more important when the disease and symptoms progress and would be characterized by higher and faster mediolateral oscillations. This would be due to a widespread pathology of the nervous system. Galna et al. (2013) [39] state the hypothesis that the more motor symptoms progress, the slower the postural reactions, leading to a greater imbalance.

In addition, the Unified Parkinson Disease Rating Scale (UPDRS), including the Hoehn and Yahr stage (UPDRS V), was used by many studies to determine the severity and the stage of disease progression [31,32,40,41,43]. From the 10 studies, three studies used at least one section of the UPDRS to quantify the severity of symptoms. However, only one completed all questionnaires. Tassoreli et al. [31] observed that the UPDRS score (mean = 30.8) was negatively correlated to the result of the Berg test and of the functional independence measure. They also observed a positive correlation between UPDRS and Fall Efficacy Scale. Viitasalo et al. [32] demonstrated that a UPDRS motor examination score above 10 corresponds to higher and faster oscillations compared to group control and patients with UPDRS motor examination score > 10. This is also true for the Parkinson’s disease group with a score over 25 [32]. Indeed, presence of a fall in the last year and presence of freezing were associated to a UPDRS score higher [43]. Hoehn and Yahr’s stage alone also had a significant impact on velocity, length, and area of postural oscillations during opened eyes tasks [32]. These results supports the impact of the severity and the stage of Parkinson’s disease on postural control. 

According to several studies, the presence of a cognitive impairment in an individual with Parkinson’s disease increases the risk of falls [12,19,41,43]. For example, the mean time to perform TUG in Parkinson’s patients with dementia is significantly increased [42]. In addition, postural instability during a cognitive dual task in PD patients with cognitive impairment is greater than in PD patients without cognitive impairment or in the healthy elderly population [43]. Although the present study would concentrate on sex differences, it must be remembered that balance deficits in this population are more likely to be linked to dynamic and anticipatory postural adjustments during functional tasks, mainly when cognitive status is affected [41,42,43]. In short, the presence of cognitive impairment negatively impacts postural balance and increases the risk of falls in individuals with Parkinson’s [12,19,41,43].

Considering Parkinson’s disease and its deficiencies, most of the pharmacologic treatments include L-Dopa to ease motor symptoms. Indeed, several studies showed positive results with PD patients under L-Dopa treatments [50]. A study of Fahn et al. suggest that levodopa medication has prolonged effect on symptoms of Parkinson’s disease [51]. A recent study of Fowler et al. (2018) evaluate the effect of levodopa response on gait and balance of PD patients. They evaluated patients using the Mobility Lab System, equipped with sensors, to compare medications ON and OFF states. Results showed improvements in gait and balance such as speed, foot strike and stride length, but also, lumbar and trunk range of motion in balance [52].

### 4.2. Comparison between Sexes

Only two studies out of 10 assessed the two sexes from impact of Parkinson’s disease on balance. According to their brief investigation on the comparison between men and women, Viitasalo et al. [32] as well as Tassorelli et al. [31] demonstrated no statistically significant results between men and women. According to QATSDD, the quality of these studies is low to moderate (21/42 and 17/42), respectively. However, according to the results of Tassorelli et al. [31], women with Parkinson’s demonstrated a lower level of functional independence and of Berg Balance Scale score than men, as well as a greater fear of falling. The authors suggest that the difference between men and women is probably due to the phenotypical expression of the disease. Women presented the tremor-dominant phenotype, which affected the results of study. They also demonstrated a positive correlation between female sex and the frequency of falls. In fact, older women tend to fall more frequently than older men, which can lead to a greater fear of falling, as well as a decreased level of functional independence [53]. This tendency could be explained by a higher level of frailty in women compared to men, predisposing them to a higher risk of falling [54]. On the contrary, some studies have shown that older women have better postural control than older men when using a gold standard measure of balance such as force platform measures [54,55]. Apparently, women perform better than men in static balance tests under force platform measures. This could be due to the integration of the systems responsible for maintaining balance (vestibular, proprioceptive, and visual) which would be different in men and women. Women would be better at integrating information from different systems to maintain balance, while men perform better in dynamic balance tests compared to women, due in part to their often higher strength and muscle mass [33]. Therefore, more studies are needed to better distinguish the differences between sexes on postural control mainly in older people with Parkinson’s disease. Unfortunately, the conclusions from only two studies are very limited. 

## 5. Conclusions

This review identified that older people with Parkinson’s have poor postural control through greater postural oscillations than those without the disease. The impact of sex should be further explored. The main clinical application of these results is that the interpretation of an evaluation of postural control on a force platform would be generalized for both sexes. However, more studies are needed to better answer this relevant research question on rehabilitation for older individuals with Parkinson’s when sex is of concern.

## Figures and Tables

**Table 1:** Inclusion and exclusion criteria.

	Inclusion Criteria	Exclusion Criteria
Main features	○ English or French	○ Other language ○ Text not available in full version
Participants	○ Mean age >65 years old ○ Human subjects ○ Presence of men and women, regardless of the proportion ○ Parkinson’s diagnosis established, any Hoehn and Yahr’s stage included	○ Mean age <65 years
Interventions/measures	○ Objective assessment of postural stability, both static and dynamic ○ With or without intervention	○ Subjective assessment of postural stability
Quote	○ All types of quotes	

**Table 2:** List of abbreviations.

Abbreviations	Definitions
BBS	BERG Balance Scale
BTD	Bone Density
COM	Center of Mass
COP	Center of Pressure
DLB (LBD)	Dementia with Lewy bodies (Lewy body Dementia)
FES	Fall Efficacy Scale
F8W test	Figure of 8 Walk Test
FIM	Functional Independence Measure
H&Y stage	Hoehn and Yahr’s Stage
MMSE	Mini-Mental State Examination
n/a	Not Applicable
ND	Not Determined
N.E.	Not Evaluated
PD	Parkinson’s Disease
PRISMA	Preferred Reporting Items for Systematic Reviews and Meta-Analyses
QATSDD	Quality Assessment Tool for Studies with Diverse Designs
TMT	Tinetti Mobility Test
TUG	Timed Up and Go
UBPI	Ultrasound Bone Profile Index
UPDRS	Unified Parkinson’s Disease Rating Scale
YO	Eyes opened
YF	Eyes closed

**Table 3:** Table from Quality Assessment Tool for Studies with Diverse Designs (QATSDD).

Score (0–3)										
Criteria	Paper: Fritz et al. (2016)	Paper: Tassorelli et al. (2016)	Paper: Galna et al. (2013)	Paper: Hiorth et al. (2013)	Paper: Galna et al. (2010)	Paper: Latt et al. (2009)	Paper: Rochester et al. (2004)	Paper: Marchese et al. (2003)	Paper: Viitasalo et al. (2002)	Paper: Morris et al. (2000)
Explicit theoretical framework	2	1	2	2	3	2	2	2	3	3
Statement of aims/objectives in main body of report	3	2	3	3	3	3	3	2	3	3
Clear description of research setting	2	2	2	2	3	2	3	2	3	2
Evidence of sample size considered in terms of analysis	0	0	0	0	0	3	0	0	0	0
Representative sample of target group of a reasonable size	1	1	1	2	1	2	1	1	1	1
Description of procedure for data collection	2	2	3	2	2	3	2	2	2	2
Rationale for choice of data collection tool(s)	2	0	0	0	2	2	2	1	3	3
Detailed recruitment data	2	2	2	1	2	2	2	2	1	2
Statistical assessment of reliability and validity of measurement tool(s) (Quantitative only)	1	0	0	0	1	0	1	0	0	0
Fit between stated research question and method of data collection(Quantitative only)	3	2	3	2	3	2	3	2	3	2
Fit between stated research question and format and content of data collection tool e.g., interview schedule (Qualitative only)	N.E.	N.E.	N.E.	N.E.	N.E.	N.E.	N.E.	N.E.	N.E.	N.E.
Fit between research question and method of analysis (Quantitative only)	2	2	2	2	3	2	3	3	2	2
Good justification for analytic method selected	1	1	1	2	2	3	2	2	0	2
Assessment of reliability of analytic process (Qualitative only)	N.E.	N.E.	N.E.	N.E.	N.E.	N.E.	N.E.	N.E.	N.E.	N.E.
Evidence of user involvement in design	0	0	0	0	0	0	0	0	0	0
Strengths and limitations critically discussed	1	2	1	2	0	2	2	0	0	1
Total (/42)	22	17	20	20	25	28	26	19	21	23

N.E., not evaluated.

**Table 4:** Characteristics of participants and measurements.

Studies	N	Mean Age ± SD (Years)	Gender (M/W)	H&Y Stage	Mean Time Since Parkinson’s Diagnosis ± SD (Years)	N Fallers (M/W)	Mean Total Score MMSE ± SD	Mean Dose L-Dopa (mg)	Dyskinesia Freezing	Balance
1. Fritz et al. 2016	Parkinson: 21	72.38 ± 4.72	13/8	ND	ND	ND	27.81 ± 1.36	ND	ND	ND
	Parkinson with dementia: 10	74.2 ± 5.16	7/3	ND	ND	ND	27.6 ± 2.51	ND	ND	ND
	Alzheimer: 21	75.05 ± 4.96	13/8	n/a	n/a	ND	22.43 ± 4.25	n/a	n/a	ND
	LBD: 21	73.95 ± 4.78	13/8	n/a	n/a	ND	22.57 ± 3.57	n/a	n/a	ND
	DLB: 11	73.7 ± 4.59	6/5	n/a	n/a	ND	24.45 ± 4.46	n/a	n/a	ND
2. Tassorelli et al. 2016	Parkinson: 42	74.28 ± 6.8	18/24	4 (stage 1) 20 (stage 2) 18 (stage 3)	8.5 ± 5.5	27 (7/20)	>24	ND	ND	Mean score BBS ± SD: 47.9 ± 12.9
	Control: 21	75.2 ± 6.5	11/10	n/a	n/a	n/a	ND	ND	n/a	ND
3. Galna et al. 2010	Parkinson: 20	65.6 ± 7.7	16/4	Stages 1 à 3	ND	ND	28.1 ± 1.5	662.5	Dyskinesia: *n* = 0 Freezing: *n* = 0	ND
	Control: 20	65.3 ± 8.0	16/4	n/a	n/a	ND	28.6 ± 1.6	n/a	n/a	ND
4. Galna et al. 2013	Same to Galna et al. 2010 study									
5. Hiorth et al. 2013	Parkinson 1: 232	73.5 ± 8.5	113/119	Mean 2.8 ± 1.1	8.6 ± 5.7	103	24.3 ± 6.8 Including *n* = 61 avec dementia	479 ± 254	ND	ND
	Parkinson 2: 207	67.9 ± 9.2	122/85	Mean 1.9 ± 0.6	2.3 ± 1.8	34	27.7 ± 2.5	n/a	ND	ND
	Control: 175	67.6 ± 9.1	104/71	n/a	n/a	3	28.5 ± 1.5	n/a	n/a	ND
6. Latt et al. 2009	Parkinson falling: 51	68.3 ± 2.1	29/22	7 (stage 1) 11 (stage 2) 33 (stage 3)	<2: *n* = 18 2–4: *n* = 13 5–7: *n* = 12 >7: *n* = 8	40	>24 (including *n* = 25 pour ≤ 27)	<500: 8 500–750: 18> 750:25	Dyskinesia: *n* = 15 Freezing: *n* = 31	Ø Walking aid Mean TUG score ± SD: 12 ± 3 s
	Parkinson’s non-falling: 62	64.4 ± 2.7	35/27	32 (stage 1) 22 (stage 2) 8 (stage 3)	<2: *n* = 33 2–4: *n* = 15 5–7: *n* = 9 >7: *n* = 5	21		<500: 16 500–750: 33>750: 13	Dyskinesia: *n* = 17 Freezing: *n* = 13	Ø Walking aid Mean TUG score ± SD: 9 ± 1 s
7. Rochester et al. 2004	Parkinson: 20	64.6 ± 7.96	12/8	Mean 2.7 ± 0.69	10 ± 6.2	ND	27.15 ± 1.98	ND	Dyskinesia: ND Freezing gait questionnaire/24: 10.7 ± 6.23	Mean score BBS ± SD: 49.4 ± 6.33
	Control: 10	63.5 ± 7.03	6/4	n/a	n/a	ND	28.9 ± 0.73	n/a	n/a	Mean score BBS ± SD: 55.9 ± 0.31
8. Marchese et al. 2002	Parkinson: 24	66.4 ± 7.9	16/8	Mean 2.5	106.8 ± 39.8 months	ND	>26	ND (all with L-Dopa)	Dyskinesia: *n* = 0 Freezing: ND	ND
	Control: 20	60.9 ± 7.4	13/7	n/a	n/a	ND	ND	n/a	n/a	ND
9. Viitasalo et al. 2002	Parkinson: 28	64.9	14/14	Mean 2.4 ± 0.8	Mean: 7.1 ≤5: *n* = 18 >5: *n* = 10	ND	ND	ND (all with L-Dopa)	Dyskinesia: *n* = 14 Freezing: ND	ND
	Control: 32	63.1	16/16	n/a	n/a	ND	ND	n/a	n/a	ND
10. Morris et al. 2000	Parkinson falling: 15	67.5 ± 8.6	7/8	>4	12.3 ± 4.9	15	ND	ND	ND	Ø Walking aid
	Parkinson non-falling: 15	68.5 ± 7.2	7/8	>4	7.5 ± 5.6	0	ND	ND	ND	Ø Walking aid
	Control: 15	68.6 ± 7.7	7/8	n/a	n/a	ND	ND	ND	n/a	Ø Walking aid

PD: Parkinson’s disease; DLB: dementia with Lewy bodies; LBD: Lewy body dementia, UPDRS: Unified Parkinson’s Disease Rating Scale; BBS: Berg Balance Scale; TUG: time up and go; MMSE: Mini-Mental State Examination; H&Y stage: Hoehn and Yahr’s stage; ND: not determined; n/a: not applicable.

**Table 5:** Summary of general results from studies.

Article	Groups	Measures	Objective	Resume
1. Fritz et al. 2016	Gr. 1: Lewy body Dementia (LBD) Gr. 2: Alzheimer (AD) Gr. 3: Parkinson (PD) Gr. 4: Dementia + Lewy bodies (DLB) Gr. 5: Parkinson + dementia (PDD)	Gait analysis with GAITrite Tests: Tinetti mobility test (TMT), Berg Balance Scale (BBS), Timed Up and Go (TUG), Figure of 8 Walk Test (F8W test).	To compare the motor function of different neurodegenerative diseases	LBD vs. PD: For LBD, ↓ speed, ↓ step length and ↑ support time. ↑ variability of spatial parameters at high speed. ↓ balance: TMT, BBS, TUG and cognitive TUG, F8W. AD vs. PD: For AD, ↑ walking speed, ↑ step length, ↑ time in the oscillatory phase of walking, ↑ performance in TMT, ↓ motor impacts, (advanced motor impacts in PD and more pronounced in LBD). PDD vs. DLB: For PDD, ↓ TMT performance and ↓ oscillation time during backward walking indicates that PDD: ↓ dynamic and anticipatory balance during functional activities. For LBD and PD, ↑ performance in TUG, cognitive TUG, TMT, BBS and F8W is associated with better gait parameters.
2. Tassorelli et al. 2017	Gr. 1: PD Gr. 2: control	Bone density (BTD), Ultrasound Bone Profile Index (UBPI), stabilometric platform, functional independence measure (FIM), UPDRS, BBS, falls efficacy scale (FES), blood test, number of falls in the last year and reading of the medical file.	Assessment of clinical and biomechanical characteristics associated with falls, fractures and bone health in the PD population.	Stabilometry: ↑ length of the oscillations. No difference according to sex, age, stage of disease and UPDRS. Female PD vs. Male PD: For women, ↓ FIM, ↓ balance, ↑ fear of falling, ↑ falls. For men and women, the same risk of fracture among PD fallers. Age-associated and sex-associated comparison: ↓ bone density and ↑ postural instability. For PD, ↑ FIM and ↓ instabilities correlate with better bone health.
3. Galna et al. 2013	Gr. 1: PD Gr. 2: control	Center of mass (CoM) and center of pressure (COP) displacement parameters calculated by the VICON system	Find out if people with Parkinson’s (mild to moderate severity) have abnormal disruption of the center of mass as a result of stepping over obstacles while walking on level ground.	PD vs. Control: For PD, ↑ 21% lateral sway (↑ 12% at equal walking speed), ↑ lateral sway speed (↑ over obstacle). ↑ 13% incline angle before step-over, ↑ CoM medial vs. CoP during step-over. In both groups, ↑ medio-lateral sway while crossing over an obstacle compared to flat terrain. For PD, ↑ medial sway associated with ↑ motor severity when the foot straddling the obstacle is over the obstacle. ↑ swing speed with ↑ PD severity.
4. Hiorth et al. 2013	Gr. 1: control Gr. 2: PD	UPDRS Fall frequency during the year	To examine the clinical characteristics of falling and non-falling patients in a clientele with different stages of Parkinson’s disease.	An elevated UPDRS activity of daily living and UPDRS therapy complication score as well as the presence of postural instability and gait instability increase the risk of falling.
5. Galna et al. 2010	Gr. 1: control Gr. 2: PD	Foot movement parameters with the VICON motion analysis system when climbing over an obstacle	To investigate the effect of Parkinson’s disease on the walking trajectory and on the adaptation of the spatiotemporal gait when approaching and stepping over an obstacle on the ground.	Patient PD: ↓ walking speed, ↓ step length and ↑ time in bipodal position when compared with control.
6. Latt et al. 2009	Gr. 1: PD falling Gr. 2: PD non-falling	Timed Up and Go (TUG), UPDRS, H & Y, fall history.	To design a fall risk screening tool using measurement tools used in the clinic and to design a fall risk assessment tool to guide fall prevention interventions.	Indicators of a risk of falls: Frontal Assessment Battery ≤ 17/18 ↓ TUG score ↓ UPDRS score ↑ H & Y score Fall in the last year
7. Rochester et al. 2004	Gr. 1: control Gr. 2: PD	Analysis of the approach with the VAM (Vitaport Activity Monitor) during different tasks.	To assess the interference on walking of functional activities at home of people with Parkinson’s and the contribution of clinical symptoms on walking disorders.	Walking speed ↓ in the PD group (26.5% reduction). Speed significantly ↓ in the PD group during cognitive dual-task and multitasking, but not during dual motor task. Additionally, step length ↓ in the PD group compared to the control group. ↓ stride length in the PD group during cognitive dual task and multitasking and in the control group during multitasking. No difference between groups for step frequency.
8. Marchese et al. 2003	Gr. 1: control Gr. 2: PD	Force platform on 3 conditions: Basic, counting down and with a motor task. Each condition was performed with eyes open [2] and closed (YF).	To analyze the effect of concurrent motor or cognitive tasks on balance control.	No difference between the groups for the basic condition (both YO and YF), more variations in the PD group (YO and YF) than in the control group during the countdown and the concomitant motor task.
9. Viitasalo et al. 2002	Gr. 1: control Gr. 2: PD	Postural swing by inclinometry.	To test a new method of assessing balance control in people with Parkinson’s.	↑ sway in group PD vs. control. Swing speed influenced by stage of disease (UPDRS) and time since disease onset. Age or gender does not matter.
10. Morris et al. 2000	Gr. 1: control Gr. 2: PD non-falling Gr. 3: PD falling	Time held in different conditions (separate feet, tandem, etc.).	To determine the effect of double tasking on postural instability in people with Idiopathic Parkinson’s compared to healthy subjects.	Group control holds 30 s. for all positions even in double task. Group Non-falling PD and falling PD did not hold the 30 s in the tandem and one leg stance positions. Some people from group PD falling could not hold in tandem or one leg stance. In a double task, the falling and non-falling PD groups did not last 30 s. in the step, tandem and one leg stance positions.

PD: Parkinson’s disease; DLB: dementia with Lewy bodies; LBD: Lewy body dementia, UPDRS: Unified Parkinson’s Disease Rating Scale; BBS: Berg Balance Scale; TUG: time up and go; MMSE: Mini-Mental State Exam.
